# Confocal endomicroscopy diagnostic criteria for early signet-ring cell carcinoma in hereditary diffuse gastric cancer

**DOI:** 10.1186/s12876-023-02822-3

**Published:** 2023-05-23

**Authors:** Nastazja D. Pilonis, Maria O’Donovan, Susan Richardson, Rebecca C. Fitzgerald, Massimiliano di Pietro

**Affiliations:** 1 Early Cancer Institute, University of Cambridge, Cambridge, CB2 0XZ UK; 2 Department of Gastroenterology, Hepatology and Clinical Oncology, Medical Centre for Postgraduate Education, Warsaw, Poland; 3 Department of Histopathology, Addenbrooke’s Hospital, Cambridge, UK

**Keywords:** Hereditary diffuse gastric cancer, CDH1, Confocal laser endomicroscopy, Endoscopic surveillance, Signet ring cell carcinoma

## Abstract

**Background:**

Recognition of early signet-ring cell carcinoma (SRCC) in patients with hereditary diffuse gastric cancer (HDGC) undergoing endoscopic surveillance is challenging. We hypothesized that probe-based confocal laser endomicroscopy (pCLE) might help diagnose early cancerous lesions in the context of HDGC. The aim of this study was to identify pCLE diagnostic criteria for early SRCC.

**Methods:**

Patients with HDGC syndrome were prospectively recruited and pCLE assessment was performed on areas suspicious for early SRCC and control regions during an endoscopic surveillance procedure. Targeted biopsies were taken for gold standard histologic assessment. In Phase I two investigators assessed video sequences off-line to identify pCLE features related to SRCC. In Phase II pCLE diagnostic criteria were evaluated in an independent video set by the investigators blinded to the histologic diagnosis. Sensitivity, specificity, accuracy, and interobserver agreement were calculated.

**Results:**

Forty-two video sequences from 16 HDGC patients were included in Phase I. Four pCLE patterns associated to SRCC histologic features were identified: (A) glands with attenuated margins, (B) glands with spiculated or irregular shape, (C) heterogenous granular stroma with sparse glands, (D) enlarged vessels with tortuous shape. In Phase II, 38 video sequences from 15 patients were assessed. Criteria A and B and C had the highest diagnostic accuracy, with a κ for interobserver agreement ranging from 0.153 to 0.565. A panel comprising these 3 criteria with a cut-off of at least one positive criterion had a sensitivity of 80.9% (95%CI:58.1—94.5%) and a specificity of 70.6% (95%CI:44.0—89.7%) for a diagnosis of SRCC.

**Conclusions:**

We have generated and validated off-line pCLE criteria for early SRCC. Future real-time validation of these criteria is required.

## Introduction

Hereditary diffuse gastric cancer (HDGC) is a cancer syndrome associated with early onset diffuse gastric cancer (DGC) and lobular breast cancer (LBC) [1]. HDGC is linked to germline pathogenic variants (PV) in the *E-cadherin* gene (*CDH1*) that are inherited in an autosomal dominant pattern; however, in approximately 70% of families with DGC clustering a genetic cause cannot be identified [2, 3, 4]. The lifetime risk of DGC in individuals who fulfill HDGC criteria is approximately 70% for men and 56% for women [5].

Due to the high penetrance of DGC, individuals with *CDH1*-PV are recommended to undergo prophylactic total gastrectomy (PTG) [6]. However, *CDH1*-PV carriers who have comorbidities or refuse or wish to delay gastrectomy due to psychological and social reasons, are offered endoscopic surveillance with the aim to detect DGC at the early stage. Endoscopic surveillance is recommended for individuals with strong family history, but no *CDH1*-PV, who should not be offered prophylactic gastrectomy in the absence of pathological findings. [6]. However, endoscopic diagnosis of early DGC is challenging because in-situ and intramucosal SRCC are often located under the epithelial surface at the bottom of the gastric crypts, and therefore often cannot be visualized even under close endoscopic mucosal evaluation performed by expert endoscopists with experience in HDGC surveillance [7]. Occasionally, characteristic pale areas containing SRCC can be seen on white light endoscopy, however the sensitivity of pale areas for early SRCCs is estimated to be less than 30% [8]. Image enhancement with narrow band imaging (NBI) helps better delineate the borders of pale areas. Moreover, NBI, in conjunction with optical magnification help differentiate pathological pale areas from mucosal scars due to previous biopsies, which also appear as whitish mucosa. We have recently showed that endoscopic criteria based on shape, borders, reproducibility and micro-structural and micro-vascular patterns of pale areas achieve a sensitivity of 67.3% and a specificity of 90.2% for early cancer diagnosis [9].  However, relying on biopsies targeted by high-definition endoscopy and NBI would lead to underdiagnosis in up to 60% of patients [10]. Therefore, to ensure optimal detection and risk stratification, an extensive mapping biopsy protocol remains an integral component of HDGC endoscopic surveillance [10]. The current recommendation is to take 30 biopsies scattered throughout the different sections of the stomach as follows: 5 biopsies in the antrum, transitional zone, fundus and cardia, and 10 in the gastric body [6]. Chromoendoscopy with Congo Red has been showed to help identify early DGC, however, concerns over potential toxicity of this agent have dampened the enthusiasm [11]. Other dyes and electronic chromoendoscopic techniques such as indigo carmine chromoendoscopy [12] and autofluorescence imaging [8], have not been proven to be useful [11] in patients with HDGC.

Probe-based confocal laser endomicroscopy (pCLE) provides microscopic views of the gastro-intestinal mucosa at cellular resolution to allow real-time diagnosis of early neoplasia [5]. Although pCLE provides point imaging and has the potential limitation of sampling error, similar to biopsy forceps, the possibility to swipe the probe across the mucosal plane allows interrogation of a larger mucosal area compared to standard biopsies. In addition, given the deep location of early SRCC within the mucosa and the penetration of pCLE view to a depth of 65 µm [13], this technique has the potential to reveal features otherwise not seen with conventional endoscopy. Therefore, the aim of this study was to identify and validate the pCLE diagnostic criteria for early SRCC in HDGC syndrome.

## Methods

### Study design

This was a prospective study in a single tertiary referral center for HDGC. Patients undergoing surveillance endoscopy received pCLE examination and video sequences were recorded. The development of the pCLE criteria was structured in two stages. In the first phase (Phase I) we aimed to define the pCLE diagnostic criteria for SRCC via off-line analysis of a first set of pCLE video sequences and matching histopathological slides. In the second phase (phase II), we aimed to validate the criteria identified in the previous stage of the study via off-line assessment of an independent set of pCLE video sequences by investigators blinded to histopathological diagnosis.

### Study population

The study population consisted of individuals recruited to the ethically approved Cambridge Familial Gastric Cancer Study (MREC 97/5/32), who were aged 18 years or older and able to provide written informed consent. Patients enrolled in endoscopic surveillance are managed by a multidisciplinary team consisting of a medical geneticist, gastroenterologists, upper gastrointestinal surgeons, psychologists, nutritionists and clinical nurse specialists. Patients are counselled about the recommendation to undergo prophylactic total gastrectomy when a *CDH1* PV is detected, and all are offered a baseline endoscopy before surgery. Those who prefer to defer surgery when a germline genetic cause if not identified or due to comorbidities or psychosocial reasons are offered endoscopic surveillance. Study endoscopies were performed either as a first baseline investigation or as follow up. All the individuals included into the study fulfilled the testing clinical criteria for HDGC. The *CDH1* status was known for all the individuals. Patients who underwent pCLE assessment as part of Phase I of the study were excluded from Phase II.

### Endoscopy procedures and histological analysis

To ensure high-quality, clear mucosal views in the stomach, all the individuals were administered orally a water solution of simethicone with N-acetylcysteine before gastroscopy as previously described [14]. All patients received intravenous sedation consisting of opioid analgesic (Fentanyl) and benzodiazepine (Midazolam). All the endoscopic procedures in the study were performed with high-definition endoscopes with available NBI and optical magnification (FQ260Z or H290Z, Olympus, Tokyo, Japan) by an endoscopist with experience in HDGC endoscopic surveillance and confocal assessment (MDP). During each procedure, gastric mucosa was assessed in the systematic approach using white light imaging (WLI) and narrow band imaging (NBI) modalities for all the anatomic regions for the identification of suspicious lesions, with particular attention to areas of whitish discoloration (pale areas), which are pathognomonic of early SRCC in the context of HDGC. Optical zoom was used at the discretion of the investigator to achieve additional information on the nature of the lesions identified. Pale areas identified using WLI and NBI were assessed with pCLE. Diathermy coagulation was performed proximally and distally to the pale areas to ensure precise positioning of the pCLE probe. pCLE assessment was performed using Cellvizio Gastroflex™ (Mauna Kea Technologies, France) after intravenous fluorescein injection (2.5 mL of 10% solution). The endoscopist was allowed to select up to 4 pale areas and one negative control region in the gastric mucosa for pCLE assessment. Targeted biopsies from these areas were taken for histologic assessment. Random biopsy specimens were taken from each of the six anatomic regions of the stomach (pre-pyloric area, antrum, transitional zone, body, fundus, and cardia). All biopsies were examined by the experienced gastrointestinal pathologist. All biopsies underwent staining with hematoxylin and eosin, as well as Periodic Acid-Schiff staining at discretion of the pathologist. Digital video and still image were captured to document confocal findings.

### Phase I: generation of pCLE diagnostic criteria for SRCC

**Fig. 1:**
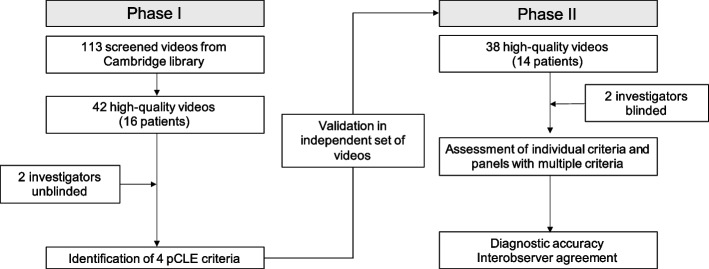
Schematic representation of study flowchart

In this part of the study, an investigator (NDP) screened a pool of 113 video clips extracted from 16 endoscopic procedures with pCLE imaging and identified 42 good quality video sequences derived pale areas and control regions (Fig. 1). The selected videos were assessed off-line by an endoscopist with extensive experience in luminal gastro-intestinal tract pCLE diagnosis (MDP) and a pathologist expert in HDGC and with research experience in pCLE (MOD). The investigators focused on the identification of pCLE features that correlated with matched histological finding of SRC in comparison with negative control cases. In this phase, the investigators had access to the digital library of the histopathological slides from all the biopsy specimens corresponding to the location of the pCLE recordings. pCLE patterns were matched with histologic features to help interpret morphology of confocal images.

### Phase II: validation of pCLE criteria for SRCC

In the second phase of the study, an independent set of 38 good quality videos from 15 patients were selected for the validation (Fig. 1). In each video sequence, the two investigators, blinded to the histologic diagnosis, independently assessed the diagnostic criteria in a binary fashion (positive/negative). During the assessment, the investigators had the possibility to pause and rewind as it is done during live pCLE examination with the trackball. Although it was sufficient for a single frame to make a call of positive criterion, it was the discretion of the investigator to evaluate the significance of this based on the quality of the video and the presence of artifacts.

### Statistical analysis

Sensitivity, specificity, accuracy, negative predictive value (NPV) and positive predictive values (PPV) were calculated as averages from all the investigators using 95% confidence intervals (CI). Sample size calculation was not performed due to exploratory nature of this study and the absence of data available to power the study. Interobserver agreement among investigators was calculated using Cohen's k statistic. For the analyses assessing the accuracy of the criteria as panel for SRCC diagnosis, a numerical cut-off of positive criteria was applied, regardless of the criteria. All analyses were performed in the R statistical environment.

## Results

### Study population

Overall, pCLE imaging was performed in 31 patients (Table 1). All of them (100%) had known *CDH1* status. Twenty-seven patients (87.0%) had an identified *CDH1* PV, and 1 patient (3.2%) was diagnosed with a *CDH1* variant of unknown significance (VUS). The majority (54.5%) of the individuals in the study cohort were female and a median age was 45 years (range 20–67).

**Table 1:** Characteristics of the study cohort

**Characteristic**	
Age median, years (IQR)	45 (20–67)
Males, n (%)	15 (48.4)
Fentanyl, μg; median (IQR)	75 (50–100)
Midazolam, mg; median (IQR)	5 (4–10)
Endoscopy purpose, n (%)	
- Index	10 (32.2)
- Surveillance	21 (67.8)
CDH1 pathogenic variant status, n (%)	
- Positive	27 (87.1)
- Variant of unknown significance	1 (3.2)
- Negative	3 (9.7)
Previous history of positive signet ring cell carcinoma finding n (%)	15 (48.4)
Ethnicity, n (%)	
- White British	14 (45.2)
- Other white	9 (29.2)
- Asian	8 (25.6)
Number of pale areas assessed with pCLE per procedure, median (IQR)	2 (1–5)
Number of control areas assessed with pCLE per procedure, median (IQR)	1 (1–2)
Number of targeted biopsies taken, median (IQR)	5 (2–11)
Pale area location, n (%)	
- pre-pyloric area	1 (1.3)
- antrum	44 (57.1)
- t-zone	3 (3.9)
- body	8 (10.4)
- fundus	21 (27.3)
Foci of signet ring cell location^a^, n (%)	
- pre-pyloric area	0 (0)
- antrum	16 (72.8)
- t-zone	0 (0)
- body	4 (18.1)
- fundus	2 (9.1)

^a^in targeted endoscopic biopsies

Foci of early SRCC were diagnosed in 14 patients, none of whom was diagnosed with cancer requiring immediate referral for oncological treatment. Among patients with positive findings, 8 cases were diagnosed with SRCC foci on targeted biopsies, with a total of 22 endoscopic areas (16 in the antrum, 4 in the gastric body and 2 in the fundus). Six patients were diagnosed with SRCC foci on random biopsies, with a total 10 positive biopsies (8 in the fundus and 2 in the gastric cardia). All cases with SRCC foci were detected in individuals with *CDH1* PV.

### Phase I

**Fig. 2:**
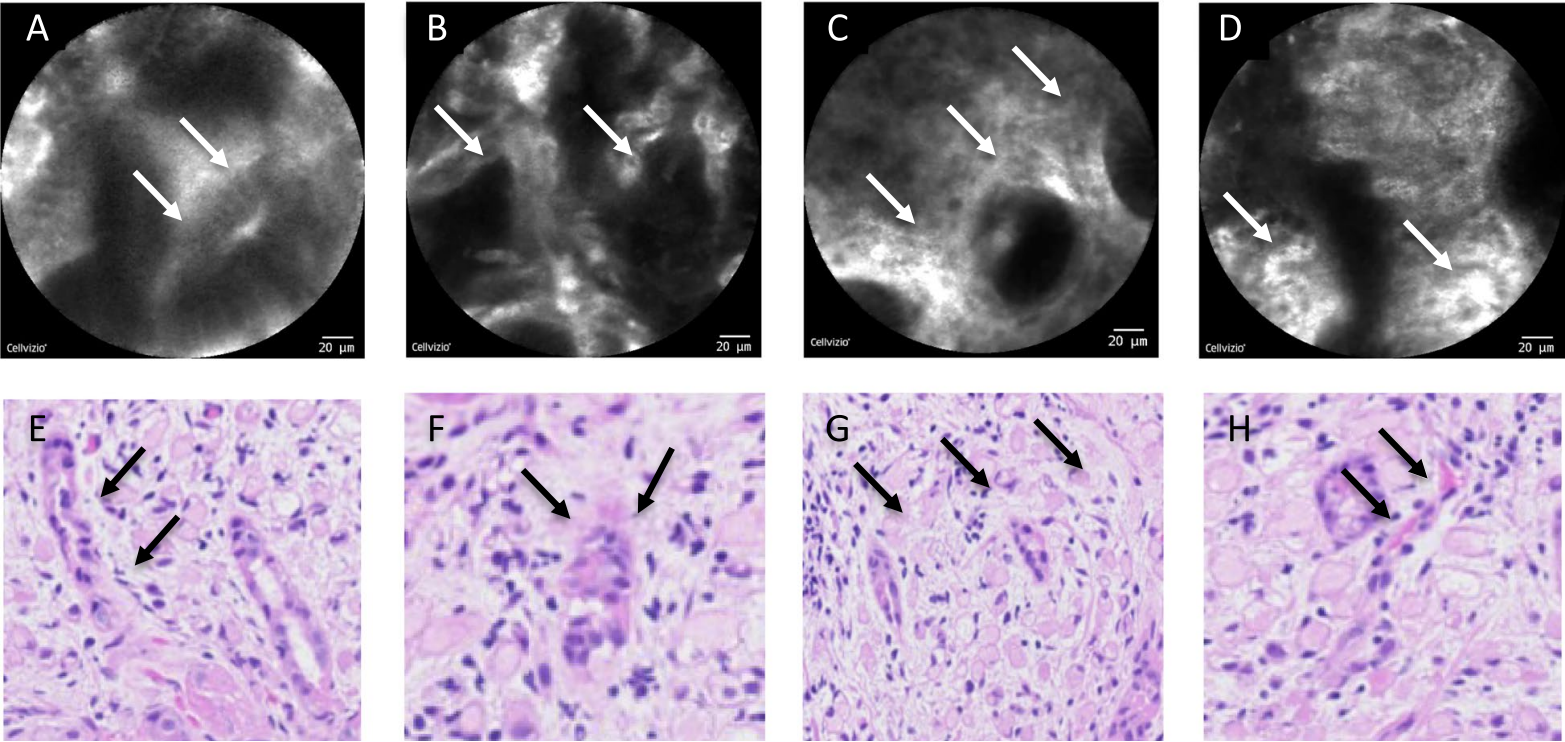
Endomicroscopic features of signet-ring cell carcinoma with histopathological correlation based on matched biopsies. **A** Glands with attenuated margins. The arrows point one of the attenuated margin of one glands. **B** Glands with spiculated or irregular shapes. The left arrow shows spiculated end of one gland and the right arrow points irregular gland shape. **C** Heterogenous granular stroma with sparse glands. The arrows show occasional glands with intermixed heterogenous stroma. **D** Enlarged vessels with tortuous shape and turbulent flow. The arrows show vessels with a visible blood flow (this criterion is best appreciated on a live dynamic view). **E** Histopathology view on H&E staining (40X) of Criterion A showing a gland with attenuated margins (arrows). **F** Histopathology view on H&E staining (40X) of Criterion B showing a gland with irregular, spiculated shape (arrows). **G** Histopathology view on H&E staining (40X) of Criterion C showing small glands spaced away by stroma infiltrated by signet ring cells. **H** Histopathology view on H&E staining (40X) of Criterion D showing showing blood vessel with irregular shape due to adjacent signet ring cells

Two investigators jointly evaluated 42 video sequences corresponding to 27 different endoscopic areas from 16 patients. Four pCLE features were identified in association with histological evidence of SRCC. These were: (A) glands with attenuated margins, (B) glands with spiculated or irregular shape, (C) heterogeneous granular stroma with sparse glands, (D) enlarged vessels with tortuous shape and turbulent flow (Fig. 2).

### Phase II

The four criteria from the phase I were assessed in an independent set of 38 videos from 14 individuals by the same 2 investigators blinded to the histologic diagnosis and any other clinical information. The overall sensitivity, specificity, accuracy, NPV, PPV and interobserver agreements for each criterion are shown in Table 2. The performance of the criterion A (glands with attenuated margins), B (glands with spiculated or irregular shape), and C (heterogenous granular stroma with sparse glands) was similar, with sensitivity ranging from 48.28% (95% CI; 29.4% to 67.5%) to 65.3% (95% CI; 43.9% to 80.1%) and specificity ranging from 85.7% (95% CI; 67.3% to 96.0%) to 89.3% (95% CI; 71.8% to 97.7%). The highest accuracy was noted for criterion C (heterogenous granular stroma with sparse glands)—74.1% (95% CI; 61.0% to 84.7). Criterion D (enlarged vessels with tortuous shape and turbulent flow) showed the lowest accuracy of 53.4% with only 20.0% sensitivity. The interobserver agreement ranged from 0.277 (criterion A) to 0.650 (criterion C).

**Table 2:** Performance of the four diagnostic criteria in the blinded validation (CI – confidence intervals)

Criterion	Average Sensitivity, %; (95%CI)	Average Specificity, %; (95%CI)	Average Accuracy, %; (95%CI)	Cohen’s Kappa	Positive predictive value, %; (95%CI)	Negative predictive value, %; (95%CI)
A. Glands with attenuated margins	50.0 (31.3–68.7)	89.3 (71.8–97.7	69.0 (55.5–80.5)	0.277	83.3 (61.8–94.0)	62.5 (53.3–80.5)
B. Glands with spiculated or irregular shapes	48.3 (29.4–67.5)	86.2 (68.3–69.1)	67.2 (53.7–79.0)	0.575	77.8 (56.7–90.4)	62.5 (53.2–70.1)
C. Heterogenous granular stroma with sparse glands	65.33 (43.9–80.1)	85.7 (67.3–96.0)	74.1 (61.0–84.7)	0.650	82.6 (64.8–92.4)	68.6 (57.1–78.1)
D. Enlarged vessels with tortous shape and turbulent flow	20.0 (7.7–38.6)	89.3 (71.8–98.0)	53.4 (39.9–66.7)	0.612	66.7 (35.6–87.9)	51.7 (38.2–65.0)

Given the sub-optimal sensitivity of individual pCLE features, we hypothesized that, when combined into a panel, the performance of the diagnostic criteria would improve. We excluded criterion D, given the very poor sensitivity. We then assessed different cut-offs for the minimum number of positive criteria to correctly diagnose SRCC. The highest average accuracy (76.3%; 95%CI 59.8%-88.6%) and best sensitivity (80.9; 95%CI 58.1–94.5) were achieved using a cut-off of 1 positive criterion, with the specificity of 70.6% (95% CI; 44.04% to 89.7%). Using cut-off of two or three positive criteria, as expected, improved the specificity but led to a drop in the sensitivity and overall accuracy (Table 3).

**Table 3:** Performance of different cut-offs on three pCLE diagnostic criteria in the blinded validation (CI confidence intervals)

Cut-off	Average Sensitivity, %; (95%CI)	Average Specificity, %; (95%CI)	Average Accuracy, %; (95%CI)	Positive predictive value, %; (95%CI)	Negative predictive value, %; (95%CI)
**≥** 1 positive criterion	80.9 (58.1—94.5)	70.6 (44.0—89.7)	76.3 (59.8—88.6)	77.3 (61.3 – 88.0)	75.0 (54.1—88.4)
**≥** 2 positive criteria	66.7 (43.0—85.4)	82.3 (56.6—96.2)	73.7 (56.9—86.6)	82.3 (61.5 -93.2)	66.7 (51.3—79.2)
3 positive criteria	40.0 (19.1—63.9)	94.44 (72.7—99.9)	65.79 (48.6—80.4)	88.9 (52.5 -98.3)	58.6 (49.3—67.3)

## Discussion

In this study, we have identified and validated pCLE diagnostic criteria to diagnose SRCC in individuals with HDGC. We have identified 3 pCLE features associated with the histological diagnosis of SRCC, which, when used as a panel, achieved a diagnostic accuracy of 76%.

Endoscopic surveillance of HDGC is recommended as alternative to surgery in patients fulfilling the HDGC criteria without a known germline mutation and in those who refuse or wish to delay risk-reducing surgery due to medical or psyco-social reasons. Although DGC has very poor prognosis and HDGC carries a 56–70% lifetime risk of gastric cancer, endoscopic surveillance with regular time intervals and strict biopsy protocol is safe in expert centres and informs the best timing of surgery [8, 10, 12, 15]. Approximately one third of patients with a *CDH1* pathogenic variant will never develop symptomatic cancer and therefore a careful watch and wait strategy is a reasonable option in some individuals especially given the profound impact of a total gastrectomy on the quality of life [16, 17]. Since endoscopic recognition of early SRCC is challenging, an intensive random biopsies protocol is recommended to obviate to the caveat of endoscopic detection. It is estimated that relying on targeted biopsies only, early SRCC can be missed in as many as 60% of patients.

One of the reasons for low sensitivity of endoscopic imaging is the location of the foci of signet ring cells deeper than the surface mucosal plane, which results in the common clinical observation of significant false negative rate of superficial biopsies even in the presence of linitis plastica [8]. Confocal endomicroscopy has the advantage of allowing deeper scanning of the gastric mucosa, which could reveal cellular and architectural irregularities otherwise missed by conventional and image enhanced endoscopy. It must be pointed out that foci of SRCC can be located deeper that the penetration of pCLE scanning. However, the pCLE criteria identified in this study do not relate to the signet ring cells per se, but rather reflect indirect effects on the tissue architecture of the SRCC foci. This is expected since the lateral resolution of pCLE does not allow direct and precise visualization of the signet-ring cancer cells. The indirect signs are due to the blurring of crypt contours due to adjacent signet ring cells (glands with attenuated margins), compression and distortion of the architecture of the crypts (glands with spiculated or irregular shape), dislocation of the glands with increased interglandular space due to clusters of signet ring cells (heterogenous granular stroma with sparse glands) and irregularities of the vasculature due to focal compression of superficial vessels with changes in the intravascular flow (enlarged vessels with tortuous shape and turbulent flow attenuated glandular margins). It is intriguing that although the irregularity in the vascular pattern is one of the most reliable features of SRCC on NBI magnification, particularly when associated with pale areas [18], the sensitivity of the criterion D was very low, suggesting that the assessment of the vasculature on pCLE remains challenging. This is in keeping with the fact that pCLE neoplastic criteria based on vessels are not included in the diagnosis of epithelial dysplasia in other organs of the GI tract [19, 20, 21, 22]. Although there is a theoretical possibility that these diagnostic criteria might relate to other gastric pathologies, such as intestinal metaplasia (IM), atrophy or H. pylori (H.p.)-related gastritis, this is unlikely to be the case. First, none of the HDGC patients included in this study presented other co-existing gastric pathologies. Second, IM, atrophy and H.p.-relted gastritis have different sets of pCLE criteria [13].pCLE has been extensively investigated in the field of intestinal type early gastric cancer [13]. However, there is scarce data on the utility for HDGC. In a previous study a systematic approach to identification of diagnostic criteria has not been used [23]. In this work the comparison between the diagnostic accuracy for early SRCC of pCLE on random locations versus the mapping biopsy protocol failed to show a significant benefit of pCLE over random biopsies. Moreover, non-targeted biopsies taken as per Cambridge protocol revealed SRCC in 11.1% of patients (4/36), whereas in-vivo assessment by pCLE showed irregular patterns in 16.7% of cases (6/36).

In our study the video sequences were obtained predominantly from pale areas identified on WLI and NBI, together with one negative control and pCLE was not used for wider interrogation of normal mucosa. The ultimate goal of utilization of pCLE as clinical adjunct would be to screen the normal looking mucosa for areas suspicious for SRCC and inform need of biopsies. For this reason, we think that a cut-off of one positive criterion is more appropriate as it is essential to optimize the sensitivity compared to specificity. Indeed, the sensitivity of pCLE (80.9%) appears to be higher than that achieved by our group using WLI and NBI magnification features (67.3%) [9]. Future studies will need to assess the diagnostic accuracy and procedural time of an imaging strategy based on more extensive pCLE assessment of gastric mucosa and targeted biopsies based on pCLE assessment versus the random biopsy protocol.

This study has several strengths. We have used a strict multi-stage process to identify and validate the pCLE criteria. The investigators have extensive experience in the field of HDGC and also worked together in the development of pCLE diagnostic criteria in different clinical applications [19]. However, there are also some limitations. Foci of SRCC can be small and can be missed by targeted biopsies, even in the presence of clearly visible pale areas. Therefore, we cannot exclude that some of the areas with no histopathological evidence of SRCC on biopsy material might indeed contain cancer due to sampling error, leading to an underestimation of the specificity. The same investigator (MDP) was involved in the endoscopic procedures and off-line assessment of the video sequences; therefore, we cannot exclude bias from recollection of the pCLE procedures findings. However, we used a wash out period of at least 8 weeks to minimise this interference and the investigator had no availability in Phase II of clinical information from the procedure making recollection of pCLE patterns extremely unlikely. The criteria were developed based on video sequences mostly derived from pale areas, therefore they might not be accurate for identification of SRCC foci located in macroscopically normal mucosa. This was necessary in order to enrich with histopathological endpoints, which are only rarely found outside pale areas and randomly distributed. Finally, these criteria have been developed in the context of HDGC and might not be applicable to diagnosis of sporadic diffuse type gastric cancer. In fact, signet ring cell lesions (insitu and pagetoid) have not been described in patients without *CDH1* PV or otherwise positive family history of DGC fulfilling clinical criteria for HDGC, therefore it is reasonable to conclude that early cancer in HDGC and sporadic setting might have difference histopathological features.

In conclusion, we have developed and validated off-line diagnostic criteria for early SRCC in the context of HDGC. The criteria identified in this study have 81% sensitivity and 71% specificity. These criteria will need to be validated in prospective studies using this technology for in-vivo diagnosis.

## Data Availability

Available to interested readers by contacting Dr. Massimiliano di Pietro at md460@cam.ac.uk.
